# Recurrence of Guillain-Barré Syndrome Is not Uncommon, and a Relapse May Require More Aggressive Treatment than the Original Condition

**DOI:** 10.5811/cpcem.63037

**Published:** 2026-07-10

**Authors:** Josef Finsterer

**Affiliations:** Neurology and Neurophysiology Center, Department of Neurology, Vienna, Austria

Dear Editor:

I read with interest the article by Vernier et al about a 67-year-old man who experienced a relapse of Miller Fisher syndrome (MFS) five years after the initial illness. The patient’s first episode resolved completely after administration of intravenous immunoglobulins (IVIG).^1^ The relapse manifested with ataxia, ophthalmoplegia, dysphonia, and gait disturbance.^1^ Despite IVIG administration, the patient did not fully recover the second time, as ophthalmoparesis and dysarthria persisted. The study is interesting but raises some points for discussion.

First, the assertion that recurrent Miller Fisher syndrome is rare is not supported by the literature. A PubMed search for “recurrent MFS” yields numerous results. Therefore, it should be concluded that relapses of Miller Fisher syndrome have been repeatedly described and are not uncommon.^2^

The second point concerns the discrepancy between the patient’s history of diplopia and ophthalmoplegia, which did not improve despite treatment, and the clinical neurological examination on admission, which did not reveal ophthalmoplegia, a key feature of Miller Fisher syndrome.^1^ This discrepancy should be resolved.

Third, the results of the investigation into the causative agents of the second episode of Miller Fisher syndrome were not reported.^1^ Although the patient had recently complained of a cough and runny nose, it was not noted whether testing for the causative agent was performed. The most common causative agents of Guillain-Barré syndrome include *Campylobacter jejuni, Haemophilus influenzae, Mycoplasma pneumoniae*, hepatitis-C virus, hepatitis-E virus, herpes simplex virus, varicella zoster virus, dengue virus, Zika virus, *Plasmodium falciparum, leptospirosis, Orientia tsutsugamushi*, and *Treponema pallidum*. In addition, infections with cytomegaly virus, HIV, hepatitis-B virus, Epstein-Barr virus, and tuberculosis, as well as recent vaccination, should be ruled out.^3^ Was severe acute respiratory syndrome-coronavirus-2 infection ruled out as a causative agent of the Miller Fisher syndrome?

Fourth, blood tests on admission showed an elevated fasting blood glucose level.^1^ Was the patient fasting before the blood draw? Was there a history of diabetes mellitus, and was the haemoglobin A1c level elevated? Since the patient did not fully recover, further investigations to clarify the underlying causes and alternative diagnoses would have been warranted. Other differential diagnoses that should have been ruled out include diabetes, myasthenia gravis, and a mitochondrial disease.

The fifth point concerns the lack of nerve conduction studies of cranial or peripheral nerves to determine whether the Guillain-Barré syndrome was an axonal or demyelinating disease. Transcranial magnetic stimulation of the facial nerve and recording of auditory- and somatosensory-evoked potentials would also have been helpful.

The sixth point relates to the discrepancy between the abstract stating that the patient had right-sided facial paralysis and the absence of facial weakness during the clinical neurological examination.^1^ This discrepancy should be investigated, and facial nerve involvement should be confirmed or ruled out.

Finally, it is unclear why the patient did not receive a second cycle of IVIGs or plasmapheresis after the first cycle proved only partially effective. Had the patient not consented to more aggressive treatment?

Before ophthalmoparesis, ataxia, sensory disturbances, gait disturbances, and dysarthria are attributed to Miller Fisher syndrome, all other differential diagnoses must be ruled out. If Miller Fisher syndrome is diagnosed, the causative factor must be identified. Once the diagnosis is confirmed, treatment should be expanded until a satisfactory recovery is achieved.
